# COVID-19 Lockdown and Consumption Patterns among Substance Use Disorder Outpatients: A Multicentre Study

**DOI:** 10.1159/000521425

**Published:** 2022-01-17

**Authors:** Lara Grau-López, Constanza Daigre, Raúl Felipe Palma-Alvarez, Marta Sorribes-Puertas, Pedro Serrano-Pérez, Marta Quesada-Franco, Lidia Segura, Marta Coronado, Josep Antoni Ramos-Quiroga, Joan Colom

**Affiliations:** ^a^Department of Psychiatry, Addiction and Dual Diagnosis Section, Hospital Universitari Vall d'Hebron, Barcelona, Spain; ^b^Psychiatry Group, Mental Health and Addiction, Vall d'Hebron Research Institute (VHIR), Barcelona, Spain; ^c^Biomedical Network Research Centre on Mental Health (CIBERSAM), Madrid, Spain; ^d^Department of Psychiatry and Forensic Medicine, Autonomous University of Barcelona, Barcelona, Spain; ^e^Subdirecció general de Drogodependències, Agència de Salut Pública de Catalunya, Barcelona, Spain

**Keywords:** Anxiety, Consumption patterns, COVID-19, Dual diagnosis, Lockdown, Pandemic, Substance use disorder

## Abstract

**Introduction:**

COVID-19 has had a great impact on mental health in the general population. However, few studies have focused on patients with substance use disorders (SUDs). This research aimed to compare the clinical status and substance use patterns of SUD outpatients, before and during confinement due to the COVID-19 pandemic.

**Methods:**

This multicentre study recruited 588 patients (29.2% women; M age = 47.4 ± 11.7 years) in thirteen centres for addiction treatment in Catalonia, Spain. All subjects were evaluated with validated instruments and ad hoc questionnaires. The sample was divided into 3 groups according to how the substance consumption pattern changed during lockdown (maintained pattern, worsened pattern, and improved pattern).

**Results:**

More than 62% of the patients maintained or worsened their consumption pattern during confinement, and about 38% improved throughout this time. Diverse factors were associated with the changes in pattern like age, addiction severity, psychological distress during lockdown, social and familial issues, and therapeutic variables.

**Conclusion:**

The home lockdown during the COVID-19 pandemic was associated with major implications for substance consumption and psychiatric distress among SUD outpatients. Considering this, the need to plan appropriate interventions in cases of similar health crises is highlighted.

## Introduction

The COVID-19 pandemic has affected the entire world [1]. Spain and also Catalonia were among the most affected areas in the first months of the pandemic, which has caused an unprecedented crisis with significant health, social, and economic repercussion [2]. To halt the expansion of COVID-19 in Spain, a nationwide at-home lockdown was required for the general population, which forced a rapid adaptation of health measures to guarantee that essential activities could continue to take place, including implementing telematic assistance, among other measures [3, 4, 5, 6].

This exceptional situation has been related to an increase in anxiety and depression in the general public [7, 8, 9], as well as in more vulnerable communities, such as patients with previous mental disorders [10, 11]. Patients with substance use disorders (SUD) often face other psychiatric pathologies at the same time, which has important connotations for the aetiology, evolution, and treatment of both disorders [12]. This co-occurrence of disorders is known as dual diagnosis, which emphasizes the interrelatedness of different disorders [13]. Dual diagnosis patients are part of a heterogeneous and complex group, with high rates of relapse and more severe symptoms of addiction [14].

Regarding substance use in the general population during lockdown, changes in drinking behaviours have been reported [15]. Two large Web-based surveys found that alcohol and tobacco use increased in this population but not use of other substances [16, 17]. One study reported that individuals consumed slightly more alcohol and also smoked marginally more cigarettes compared to the period before the lockdown [16]. Another study, that analysed changes in alcohol drinking behaviour and tobacco smoking in the general population, found that 35.5% of the study population reported an increase in drinking during the lockdown [17].

Since the beginning of the pandemic, hypotheses have been proposed on how lockdown could affect patients being treated for addictions. However, it was unknown how this historic health and social milestone would affect this vulnerable population. On one hand, it was proposed that, as had occurred in previous pandemics, substance use would increase [18] or by not being able to access usual treatments [19]. On the other hand, it was hypothesized that the decreased availability of substances may lessen their consumption [18].

Most published studies to date suggest there was an increase in substance use among SUD patients during the first months of the COVID-19 pandemic, especially in the use of legal substances. One study, carried out with alcohol-dependent patients, found an increase in alcohol consumption [20]. Another study, using hair analysis, conducted on 30 subjects, showed a decrease in the consumption of illegal drugs but a significant increase in consumption of benzodiazepines and alcohol, substances which are more easily accessible [21]. These data were confirmed in a separate study from Spain which found an increase in alcohol and benzodiazepine consumption in drug-dependent individuals [5]. Few studies include illegal substance use among patients with SUD. In the USA, a significant increase in illicit drug use (cocaine, fentanyl, heroin, and methamphetamine) was observed through positive urine drug tests in a population diagnosed with, or at risk for, SUD during the 4 months after the COVID-19 pandemic declaration [22]. An increase in overdoses has also been described in the emergency departments in the USA during the pandemic [23]. However, despite these studies, there are little data on the impact of lockdown on patients being treated for SUD, especially those who consume illegal drugs.

In terms of COVID-19 infection, SUD patients were considered an at-risk group [24]. Diverse publications have warned about greater contagion risk and physical consequences among those with ill health and a poor social situation [25, 26, 27]. Nonetheless, similar incidence of severe COVID-19 effects has been reported for SUD patients and for people without SUD [5, 26, 28], although studies are needed to analyse the risk of COVID-19 infection in SUD population.

This study aimed to compare the clinical status and substance use patterns, before and during confinement due to the COVID-19 pandemic, in SUD patients treated in outpatient centres for drug addiction in Catalonia. An analysis was also done of clinical- and COVID-19-related variables in patients that improved, maintained, or worsened their consumption pattern during confinement compared to their status before.

## Materials and Methods

### Study Design and Patients

A multicentre, descriptive study was conducted on SUD patients who received treatment in outpatient centres for drug addiction in Catalonia. Thirteen centres participated in the study, and they collected retrospective information about clinical features and substance use information during the 3-month lockdown, from March 15, 2020, to May 24, 2020. Recruitment lasted from March 2020 to September 2020. The inclusion criteria were people with a diagnosis of SUD aged older than 18 years. Patients with low Spanish proficiency that interfered with their ability to understand the study proposal were excluded. The project was approved by the Ethics Committee of Vall d'Hebron Hospital (PR-(AG)386–2020). The legal department of the Sub-Directorate General of Drug Dependence of Health/Public Health Agency of Catalonia approved the database. Patients did not receive any financial compensation, and written informed consent was obtained from all participants. In the case of telephone visits, consent was procured orally and ratified in writing in the following in-person visit.

### Procedure

After obtaining written informed consent from the participants, the psychiatrists or psychologists in charge of each participant interviewed them using an ad hoc questionnaire to evaluate their clinical and substance use status both before and during the lockdown caused by the COVID-19 pandemic. The interviews were devised through either in-person visits or telematically during the patient's usual programmed follow-up visits. All the information was entered into a database following the anonymization of patients and professionals. Vall d'Hebron Hospital and the Sub-Directorate General of Drug Dependence of Health/Public Health Agency of Catalonia coordinated the study and the data collection.

### Instruments and Variables

#### Previous Sociodemographic and Clinical Features

An ad hoc designed interview was used to evaluate demographic and clinical data at the time of enrolment. Sociodemographic features recorded were gender, age, nationality, civil status, housing, educational level, employment status, and criminal record. Information regarding SUD included primary diagnosis, whether there was polysubstance use (understood as 3 or more SUDs), drug administration route, previous SUD treatments, and length of abstinence prior to lockdown and the time of abstinence at the beginning of lockdown. Dual diagnosis was assessed by a trained psychiatrist or clinical psychologist and established by clinical judgement, following the DSM-5 criteria. After dual diagnosis assessment, the mental disorders diagnosed were grouped by psychotic, depressive, anxiety, and personality disorders.

#### Substance Use Status, COVID-19-Related Variables, and Psychopathological Variables during Lockdown

Substance use pattern during the lockdown was assessed by the same ad hoc designed questionnaire. This questionnaire included the evaluation of SUDs before and during the lockdown, history of SUD, changes in substance consumption during lockdown, clinical variables regarding psychiatric status, and COVID-19-related variables (described in Tables 1, 2). Considering changes in substance consumption (in terms of the amount consumed, relapses or abstinence) during the lockdown (compared to before), participants were classified into 3 groups: maintained pattern (patients whose consumption remained the same in terms of amount, frequency, and type of consumed substance), worsened pattern (patients who relapsed in their use of the main or another substance or increased consumption, with the exception of tobacco), and improved pattern (patients who achieved or maintained abstinence or those who consumed less frequently or in lower amounts compared to previous substance consumption). The types of substances consumed during the lockdown and craving levels were also recorded.

COVID-19-related variables were measured, including variables such as concern due to COVID-19, COVID-19 symptoms, confirmed COVID-19 infection, hospitalization due to COVID-19, breaking lockdown norms, working during lockdown, decreased income because of the pandemic, changes in housing status, family relationships, social matters, and health status. Also recorded was whether the treatment was telematic or in person and how patients valued these treatments.

Finally, psychopathological variables during lockdown were measured, such as insomnia symptoms, eating disorder symptoms, sexual alterations, and attendance at the psychiatric emergency room for SUD or other matters. Moreover, the following instruments were used to assess anxiety symptoms, general psychiatric status, and clinical global impression during lockdown:

- Overall psychiatric severity was evaluated through the Clinical Global Impression-Severity scale (CGI-S [29]) and Clinical Global Impression-Improvement scale (CGI-I [29]) which assess improvement and worsening during lockdown. They implement the widely used Likert scale (0–7 points).
- The Clinical Anxiety Scale (CAS) [30] is a hetero-applied scale which measures anxiety symptoms during lockdown using 7 items that are scored on a 5-point Likert scale (0–4). Five or more points indicate the presence of anxiety symptoms. It has adequate validity, reliability, and sensitivity to change. Assessment of anxiety symptoms was included because anxiety symptoms can be induced by the COVID-19 crisis and because they are frequently present in people with addiction.
- The Brief Psychiatric Rating Scale (BPRS) [31] assesses 18 symptom domains through clinical judgement and questioning, a Likert scale is used from 0 (not present) to 6 (extremely severe). The evaluated domains are somatic concern, anxiety, emotional withdrawal, conceptual disorganization, guilt feelings, tension, mannerisms and posturing, grandiosity, depressive mood, hostility, suspiciousness, hallucinatory behaviour, motor retardation, uncooperativeness, unusual thought content, blunted affect, excitement, and disorientation. The evaluation takes 10 min, and following the interview with the patient, the clinician rates each item on the Likert scale through observation and questioning depending on the assessed item.

### Statistical Analysis

Descriptive statistics (mean, standard deviation, and frequency tables) of the main variables were calculated. The data were then analysed at the bivariate level in order to compare the 3 groups established according to the consumption pattern during lockdown. The χ^2^ test was used to compare categorical variables and the ANOVA test for continuous variables when 3 groups were compared. The χ^2^ test was considered not applicable when one or more of the cells had an expected count <5. To reduce false-positive results, the Bonferroni correction for multiple tests was performed according to the number of tests in each bivariate analysis and to avoid type 1 error.

Only variables considered clinically relevant, those which retained statistical significance after Bonferroni correction and without multicollinearity, were included in the multinomial regression models. The compared groups by the dependent variable were improved pattern, maintenance pattern, and worsened pattern. At the bivariate level, men and women were analysed together because there were no significant differences between genders in the dependent variable; however, in the multivariate analysis, gender and age were included in the models performed. All statistical hypotheses were two-sided, and a *p* value of <0.05 was considered statistically significant. SPSS version 20 (SPSS Inc., Armonk, NY, USA) for Windows was used for all analyses.

## Results

### Sample Recruitment and Sample Features

A total of 588 patients were enrolled, representing 9.1% of the patients treated in the 13 participating centres. During the recruitment period, the patients who attended or were visited telematically were consecutively invited to participate. It is likely that due to restrictions derived from the pandemic, 38.4% were not visited during the recruitment period. Furthermore, the clinicians did not invite 16.2% of the patients to participate due to different practical reasons (e.g., limitation of time and involvement of professionals in supporting COVID-19 patients). Finally, 26.8% declined to participate in the study, and 9.5% were excluded due to a language barrier.

Each centre provided data on the patients in terms of age, sex, and substances consumed. The sociodemographic, clinical, and COVID-19-related variables both before and during lockdown in Catalonia are shown in Tables 1 and 2. Women made up 29.2% of the sample, while 70.7% were men, and the mean age was 47.4 ± 11.7 years. Regarding primary SUD diagnosis before lockdown, it was observed that alcohol and cocaine use were the most prevalent form of SUD, and 32.7% of the patients were polysubstance users. It was found that 67% of the participants had a dual diagnosis, with the most frequently co-occurring disorders being depressive disorders followed by anxiety disorders, and 20.2% had personality disorders (See Table 1). Table 2 describes variables related to SUD, COVID-19, and psychiatric status during lockdown; 25.2% of patients were classified in the maintained pattern, 36.9% in the worsened pattern, and 223 (37.9%) in the improved pattern group (157 of them maintained previous abstinence, 24 patients achieved abstinence, and 42 patients reduced substance use during lockdown). It was found that 31% of the patients with benzodiazepine use disorder (BUD) reported increased consumption of this substance, in the case of cannabis 29% reported increased use, and less frequently (12.4%) reported was increased opioid use in patients with opioid use disorder. For the entire sample, alcohol was the most common substance used, followed by cannabis and cocaine. Regarding the COVID-19-related variables, 58.2% of patients reported feeling concerned about the disease, 13.1% reported COVID-19 symptoms, and only 1.4% (*n* = 8) were infected. Around 35% of the sample acknowledged breaking the lockdown norms. Regarding psychiatric status, 60% reported anxiety symptoms, 35.4% depressive symptoms, and 40.4% reported that their psychiatric symptoms worsened during lockdown (See Table 2).

### Comparison of the Consumption Pattern before and during Lockdown

Tables 3 and 4 show the comparison of the different variables studied among the 3 groups. It was found that the patients in the worsened-pattern group were significantly younger, were mainly in treatment for cocaine use disorder, were polysubstance users, used the injected route more frequently, fewer had been abstinent for a year, and had more frequent personality disorders (See Table 3). Table 4 shows that patients whose consumption pattern worsened were more commonly seen for emergencies due to SUD, faced more withdrawal symptoms, and consumed substances more often.

Regarding COVID-19-related variables, patients whose consumption worsened reported breaking lockdown restrictions more frequently. The percentage of infection was higher among patients whose consumption patterns worsened during lockdown; however, due to the low prevalence, it is not possible to make reliable statistical comparisons. In addition, those in this group had more family conflicts and unresolved social issues. Patients in this group were treated more frequently in face-to-face meetings than those in the other groups, who had more telematic visits. Also, patients whose consumption worsened valued the treatment offered less positively. Concerning psychiatric status, it was observed that patients whose consumption pattern worsened experienced insomnia and anxiety symptoms more frequently and had higher scores in the BPRS, as well as greater severity in the CGI-S and worsened in terms of CGI-I (See Table 4).

### Multinomial Regression Models on Previous Consumption Pattern, COVID-19-Related Variables, SUD Variables, and Psychiatric Status during Lockdown

Table 5 shows multivariate analysis allowing a pairwise comparison of the groups. Model 1 analysed previous lockdown variables according to the consumption pattern (χ^2^ = 74.6, *p* < 0.0001, Negelkerke = 0.134). The variables that retained statistical significance in model 1 were age, poly-SUDs, and current cocaine use disorder.

Model 2 shows independently related variables with COVID-19 variables during lockdown (χ^2^ = 278.1, *p* < 0.0001, Nagelkerke = 0.445). All variables included in model 2, except gender, retained statistical significance (age, telematic treatment, positively valued treatment, break the lockdown norm, worsened family relationship, unresolved social matters).

Model 3 analyses the variables related to the patients' psychiatric status during lockdown (χ^2^ = 90.9, *p* < 0.0001, Nagelkerke = 0.164). The variables that retained statistical significance in model 3 were age, insomnia symptoms, anxiety symptoms, and BPRS score.

## Discussion

This study shows that lockdown in Catalonia during the first phase of the COVID-19 pandemic was associated with changes in the consumption pattern of a great part of SUD patients. Patients who worsened their consumption presented greater social difficulties and psychiatric disturbances.

Regarding coronavirus infection, 8 patients (1.4%) reported confirmed virus infection. A prior study performed in Italy reported that SUD patients are more frequently tested for COVID-19 infection, but the probability of a positive result is lower than in the general population [28]. It should be mentioned that during the study period COVID-19, tests were still widely unavailable; therefore, it is difficult to make comparisons.

This study found that about 40% of the patients worsened their consumption pattern, increasing quantity and frequency of use during the lockdown established during the first months of the COVID-19 pandemic. Due to the design of the study, it is not possible to establish causal relationships regarding the impact of confinement; however, it is of interest to compare the results with pre-pandemic studies focused on the evolution of SUD patients. Studies carried out with outpatients, by participating centres in this study, showed that after 3 months of treatment, 44.2% of the patients had relapsed [32], and 80.5% had relapsed after one year [33]. As 60% of the patients in this study consumed some legal and/or illegal substances during lockdown, it is possible to hypothesize that the confinement had a negative impact on the results of treatment. The stress produced by the health crisis, the difficulties of continuing with face-to-face treatment, and psychological distress may have affected treatment outcomes. Note that there are studies that question the effectiveness of psychosocial treatments for alcoholism and that describe high relapse rates, with treatment outcomes affected by multiple factors [34, 35]. In addition, it is possible that the patients who increased consumption had characteristics typical of addicted patients, such as difficulties in managing craving and other factors similar to the general population that contributed to increased consumption, especially of alcohol, such as using substances as coping strategies or due to boredom [36, 37] or because they perceived fewer consequences of drinking more [15]. These motivational aspects could be integrated as part of the psychological treatment.

The substances that were most consumed by patients were alcohol, cannabis, and cocaine. However, benzodiazepines, cannabis, and tobacco were the substances of which consumption increased the most compared to prior to the pandemic, among patients who previously had an addiction to these substances. A recent study reported that cannabis users who increased cannabis and tend to use cannabis to cope are at increased risk of experiencing negative cannabis-related consequences during the COVID-19 pandemic [37]. The patients with opioid use disorder were the ones who reported increased use less frequently. Around a third of patients with BUD increased the consumption of benzodiazepines, and it is possible that the COVID-19 measures and the worrying news about the consequences of the pandemic have increased the risk of using anxiolytics to face emotional distress. Therefore, it is still of interest to work on coping strategies in patients with BUD; and it should be noted that in Spain, benzodiazepines require a prescription, so most of these patients were probably seen by a specialist. In any case, benzodiazepine use should be constantly supervised in facilities where SUD patients are treated [38].

Alcohol was expected to be the most consumed substance because alcohol use disorders are the most common SUDs addressed in consultations and as its accessibility was not altered in the supermarkets during confinement. It is likely that some patients transferred their drinking in bars and restaurants to their homes, including drinking socially through digital channels; however, patients who reduced their consumption could have taken advantage of the confinement to avoid consumption. These data coincide with other Spanish studies carried out in SUD patients during this time [5, 20]. It also aligns with some studies carried out in the general population that found increased benzodiazepine and alcohol consumption [9, 39]. However, other studies conducted in general population suggest alcohol consumption declined during the first months of the pandemic [40].

Regarding demographic characteristics, there were no differences in gender according to the consumption pattern during the period studied. However, differences were observed when comparing based on age. It was observed that younger patients more frequently reported having worsened consumption. In the multivariate analyses, age was maintained as a variable independently associated with greater severity. This finding contrasts with a study conducted on patients with alcohol use disorders who attend a urine drug screening programme in Catalonia which identified that the likelihood of testing positively increased with age [20]. However, younger age has been associated with more impulsivity, and some studies have described poorer adherence and worse treatment outcomes [32, 40]. Additionally, during the pandemic, older patients may have been more careful as they identified themselves as part of the population more vulnerable to COVID-19.

When consumption pattern during lockdown was compared in terms of previous SUD and dual diagnosis, it was observed in the multivariate analysis that polysubstance users and cocaine-dependent patients had worsened consumption during lockdown. It is hypothesized that the increase in consumption in patients with these SUDs may be associated with their tendency to have a high severity of addiction and the fact that they frequently have other psychiatric disorders [41]. Furthermore, in patients with SUD, it has been described at a neurobiological level that stress is a critical determinant of relapse susceptibility, especially in subjects with a history of higher frequency cocaine use [42, 43]. Therefore, stress coping strategies should be included, especially in the treatment of cocaine SUD patients.

Polysubstance use was more frequent in the worsened-pattern group. Patients with 3 or more SUDs have a worse prognosis for the course of addiction [14], and in stressful situations, these people are likely to use substances to alleviate emotional distress caused by home confinement.

Regarding consumption patterns compared in terms of COVID-19-related variables during lockdown, it was observed at a multivariate level that telematic treatment was more frequent in the maintained-pattern group in comparison to the other groups. Further research is required to interpret these results and the role of telemedicine in addicted patients, especially in times of social and health crises. However, in this case, there was an association between face-to-face treatment and both improvement and worsening groups. It is possible that some patients had achieved improvements when undergoing in-person treatment, and on the other hand, it is probable that patients with a worse evolution had been visited in person, the telematic treatment being insufficient. Both mental health services and addiction treatment centres had to adapt to telemedicine or teletherapy during the pandemic, e.g., virtual contingency management and also online group interventions [44]. Furthermore, it is possible that individual differences and the severity of the addiction determine a patient's capacity to adapt to telematic visits as these technologies may make it more difficult to approach emotional aspects. Recently, the importance of digital inequalities has been raised, such as limited access to technology and lack of skills to leverage the technology for desirable outcomes; these factors may contribute to negative health outcomes [45]. Digital inequalities and individual features should be evaluated and considered in the treatment offered to each patient. These issues illustrate the complexity of implementing telemedicine with addicted patients and highlight the need for clinical guidelines on this issue [46].

Positive evaluation of the treatment was more frequent among patients who improved consumption. A favourable evaluation of treatment had a significant positive relationship with drug use improvement outcomes [47]. However, no study to date has examined changes in treatment patterns of individuals with SUD [27, 48], and it is expected that further research will provide information about the pros and cons of telemedicine in SUD patients.

Patients in the worsened-pattern group more frequently reported breaking the lockdown restrictions. This may be related with a greater severity of addiction, antisocial behaviours, impulsivity, less self-control, and more emotional stress [49]. On the other hand, family conflicts and unresolved social matters were independently associated with a poorer evolution of substance consumption. This relationship has been previously described [50], and during the lockdown, as previous studies reported, social and familial problems have arisen [28, 51].

The early phase of the COVID-19 pandemic has been related to an increase in depressive and anxious symptoms [7, 10]. However, no all populations have shown this impact, as an Irish study has shown [52]. Our results have shown relevant psychiatric distress in patients during confinement, with frequent symptoms of anxiety, observing that 40% of their general psychiatric status worsened. These data are consistent with other studies that have described mental health problems during the pandemic [53]. In addition, a relationship was observed between psychiatric symptoms and increased consumption during confinement. In the multivariate analysis, a higher frequency of insomnia, more anxiety symptoms, and greater symptoms measured by the general psychiatric scale were described in the worsened-pattern group. This result coincides with a study carried out in the general population that points out an increase in alcohol consumption in patients with more psychiatric difficulties [39]. Moreover, this result reinforces the association between mental distress and substance use. It is possible that substance consumption may have an anxiolytic effect and can in turn be a form of self-medication and of managing negative emotions, while conversely that substance use could also induce increased psychiatric symptoms. This highlights the need for comprehensive treatment that addresses both psychiatric symptoms and substance use, even during a health crisis.

These results should be interpreted in light of their limitations. Regrettably, we have not collected data from patients who dropped out of treatment during the study, and it was not possible to perform any analysis on these patients. Our electronic medical record system discharged 38.4% of the patients during the study period because they did not request any consultation for more than 6 months. Hence, the generalization of the results may be restricted due to the limitations of the sample. A selection effect may be present as other studies declare that patients who reduce their substance consumption are more likely to remain in treatment. The self-reported and retrospective nature of the data is another limitation because patients are less likely to admit substance consumption to their therapists, or a memory bias could exist [54]. Because of the COVID-19 measures, centres conducted urine controls tests less frequently during lockdown. Telematic treatment was not protocolized in all centres, so future studies should assess the efficacy of these interventions. Moreover, unfortunately, it was not possible to provide data from a control group; therefore, it is not possible to make causal or explanatory interpretations from the results. It has not been possible to isolate the effect of confinement from other variables, such as the evolution of patients treated for SUD. It is only possible to analyse the data at a descriptive level and to compare patient features according to the changes in their consumption pattern.

On the other hand, the strengths of this study include that it is a multicentre study where thirteen centres from Catalonia collaborated; it is important to highlight that there were no differences regarding consumption patterns among urban versus non-urban centres. The study included a large naturalistic sample, and the assessments were conducted by trained psychologists and psychiatrists specialized in addictions. Another strength of the study is that it was carried out during a critical period at a social and health level. The information provided by this research will allow the development of follow-up studies during the evolution of the pandemic.

We conclude that the home lockdown during the COVID-19 pandemic had major implications for substance use and psychiatric distress among SUD outpatients. More than 60% of the patients included in this study maintained or worsened their consumption patterns during confinement, and only 38% improved throughout this time. Diverse factors were associated with the changes in the pattern like age, addiction severity, psychological distress during lockdown, social and familial issues, and therapeutic variables. Considering this, the need to plan appropriate interventions in cases of similar health crises is highlighted.

## Statement of Ethics

This study was approved by the Ethics Committee of Vall d'Hebron Hospital (PR-(AG)386–2020). Additionally, the legal department of the Sub-Directorate General of Drug Dependence of Health/Public Health Agency of Catalonia approved the database. Written informed consent was obtained from all participants.

## Conflict of Interest Statement

Dr. Palma-Alvarez has received speaker honorariums from Angelini, Exeltis, Lundbeck, MSD, Mundipharma, and Takeda. Dr. Ramos-Quiroga has received speaker honorariums from Janssen-Cilag, Shire, Lilly, Ferrer, Medice, and Rubió. He has also received research funding from Janssen-Cilag, Lilly, Ferrer, Lundbeck, and Rubió. Dr. Grau-López has received speaker honorariums from Janssen-Cilag, Lundbeck, Servier, Otsuka, and Pfizer. All other authors report no conflicts of interest and no financial relationships with commercial interests.

## Funding Sources

The authors did not receive any funding in relation to this study.

## Author Contributions

Lara Grau-López contributed to conceptualization, methodology, validation, investigation, resources, data curation, writing original draft, and visualization. Constanza Daigre contributed to conceptualization, methodology, validation, investigation, formal analysis, resources, data curation, writing original draft, and visualization. Raúl Felipe Palma-Alvarez contributed to investigation, writing original draft, and data curation. Lidia Segura contributed to conceptualization, methodology, resources, validation, writing original draft, and visualization. Marta Coronado contributed to conceptualization, methodology, resources, validation, writing original draft, and visualization. Josep Antoni Ramos-Quiroga contributed to writing review and editing. Joan Colom contributed to conceptualization, writing review, and editing.

## Data Availability Statement

The datasets made for this study can be available on demand to the corresponding author. Part of the data could not be allowed for distribution to others than the investigation group as it may violate ethical and/or legal regulations of written consent.

## Figures and Tables

**Table 1:** Sociodemographic and clinical variables prior to lockdown in Catalonia in the total sample (n = 588)

Prior to lockdown	
Sociodemographic characteristics, *n* (%)	
Gender (men)	416 (70.7)
Mean age, years	47.4±11.7
Spanish nationality	526 (89.5)
Secondary or higher education	278 (50.5)
Marital status (married or partner)	363 (65.9)
Living with their family	399 (70.2)
Employed	149 (26.2)
Criminal records	179 (30.4)
History of SUD, *n* (%)	
Primary diagnosis	
OUD	145 (24.7)
CUD	243 (41.3)
AUD	333 (56.6)
BUD	57 (9.7)
CNUD	103 (17.5)
TUD	87 (14.8)
Poly-SUDs	204 (32.7)
Injecting drug use	94 (16)
Previous treatment for SUD	455 (74.7)
Abstinence for more than a year	188 (32)
Dual diagnosis, *n* (%)	
Dual diagnosis	394 (67)
Psychotic disorder	74 (12.6)
Depressive disorder	219 (37.2)
Anxiety disorder	129 (21.9)
ADHD	26 (4.4)
Personality disorders	119 (20.2)
Any medical condition	378 (64.3)

ADHD, attention deficit hyperactivity disorder; AUD, alcohol use disorder; CNUD, cannabis use disorder; CUD, cocaine use disorder; OUD, opiate use disorder; SUD, substance use disorder; TUD, tobacco use disorder.

**Table 2:** Clinical and COVID-19-related variables during lockdown in Catalonia in the total sample (n = 588)

During lockdown	
SUD variables, *n* (%)	
Consumption pattern evolution	
Active consumption maintained	148 (25.2)
Worsened consumption pattern	217 (36.9)
Improved consumption or abstinence	223 (37.9)
Substance use increases, *n* (%)	
Opiate use increases in OUD	18 (12.4)
Cocaine use increases in CUD	60 (24.7)
Alcohol use increases in AUD	80 (24)
Benzodiazepine use increases in BUD	18 (31.6)
Cannabis use increases in CNUD	30 (29.1)
Tobacco use increases in TUD	24 (27.6)
Substance used during lockdown, *n* (%)	
Opiate consumption	34 (5.8)
Cocaine consumption	100 (17)
Alcohol consumption	176 (29.9)
Cannabis consumption	114 (19.4)
Benzodiazepine consumption	63 (10.7)
Hospital emergency for SUD	26 (4.4)
Withdrawal symptoms	73 (12.4)
Increased craving	213 (36.2)
COVID-19-related variables, *n* (%)	
COVID-19 concern	342 (58.2)
COVID-19 symptoms	77 (13.1)
Positive COVID-19	8 (1.4)
Hospitalization because of COVID-19	2 (0.3)
Broke the lockdown norm	206 (35)
Working	100 (17)
Decreased income	170 (28.9)
Housing changed	49 (8.3)
Worsened family relationship	139 (23.7)
Unresolved social matters	80 (13.8)
Self-reported impaired health	145 (24.7)
Telematic treatment	237 (40.3)
Positively valued treatment	426 (72.4)
Psychiatric status, *n* (%)	
Insomnia symptoms	260 (44.2)
Eating disorder symptoms	286 (48.7)
Sexual behaviour alterations	85 (14.5)
Psychiatric emergencies	29 (4.9)
Anxiety symptoms (CAS)	333 (60)
Panic attacks	70 (11.9)
BPRS (above average 27)	254 (43.2)
Depressive symptoms in BPRS	208 (35.4)
CGI-S (greater severity 5–7)	69 (11.8)
Worsening global impression of change	237 (40.4)

AUD, alcohol use disorder; BPRS, Brief Psychiatric Rating Scale; BUD, benzodiazepine use disorder; CGI-S, Clinical Global Impression-Severity scale; CNUD, cannabis use disorder; CUD, cocaine use disorder; OUD, opiate use disorder; SUD, substance use disorder; TUD, tobacco use disorder.

**Table 3:** Consumption pattern during, compared to before, lockdown by sociodemographic characteristics, SUDs, and dual diagnosis

	Improved pattern (n = 223)	Maintained pattern (*n* = 148)	Worsened pattern (*n* = 217)	Statistic	*p* value
Sociodemographic characteristics, *n* (%)					
Gender (men)	154 (69.1)	104 (70.3)	158 (72.8)	0.8	0.68
Mean age, years	49.6±12.2	48.7±11.6	44.3±10.6	13.2	0.0001*
Spanish nationality	199 (89.2)	135 (91.2)	192 (88.5)	0.7	0.69
Secondary or higher education	119 (54.8)	63 (44.7)	96 (49.7)	3.6	0.166
Marital status (married or partner)	146 (67.3)	102 (72.3)	115 (59.6)	6.2	0.045
Living with their family	155 (71.4)	110 (75.9)	134 (60)	5.0	0.082
Employed	51 (23.5)	41 (28.3)	57 (27.7)	1.4	0.504
Criminal record	55 (24.7)	45 (30.4)	79 (36.4)	7.2	0.028
SUDs, *n* (%)					
Primary diagnosis					
OUD	39 (17.5)	41 (27.7)	65 (30)	10.2	0.006
CUD	65 (29.1)	50 (33.8)	128 (59)	45.0	0.0001 *
AUD	135 (60.5)	79 (53.4)	119 (54.8)	2.3	0.315
BUDs	11 (4.9)	14 (9.5)	32 (14.7)	12.1	0.002
CNUD	28 (12.6)	29 (19.6)	46 (21.2)	6.3	0.043
TUD	34 (15.2)	15 (10.1)	37 (17.5)	3.9	0.145
Poly-SUDs	44 (19.7)	56 (37.8)	104 (47.9)	39.5	0.0001 *
Injecting drug use	20 (9)	24 (16.2)	50 (23)	16.2	0.0001 *
Previous treatment for SUD	171 (76.7)	114 (77)	170 (78.3)	0.2	0.911
Abstinence for more than a year	93 (41.7)	61 (41.2)	34 (15.7)	42.1	0.0001 *
Dual diagnosis, *n* (%)					
Dual diagnosis	155 (69.5)	91 (61.5)	148 (68.2)	2.8	0.245
Psychotic disorder	27 (12.1)	19 (12.8)	28 (12.9)	0.1	0.963
Depressive disorder	92 (41.3)	49 (33.1)	78 (35.9)	2.8	0.25
Anxiety disorder	51 (22.9)	22 (14.9)	56 (25.8)	6.3	0.042
ADHD	9 (4)	6 (4.1)	11 (5.1)	0.3	0.843
Personality disorders	33 (14.8)	21 (14.2)	65 (30)	20.1	0.0001 *
Any medical condition	151 (67.7)	95 (64.2)	132 (60.8)	2.3	0.321

ADHD, attention deficit hyperactivity disorder; AUD, alcohol use disorder; BUD, benzodiazepine use disorder; CNUD, cannabis use disorder; CUD, cocaine use disorder; OUD, opiate use disorder; SUD, substance use disorder; TUD, tobacco use disorder.

* Significant after Bonferroni correction.

**Table 4:** Consumption pattern during, compared to before, lockdown by SUD variables, COVID-19-related variables, and psychiatric status during lockdown

	Improved pattern (n = 223)	Maintained pattern (n = 148)	Worsened pattern (*n* = 217)	Statistic	*p* value
SUD variables, *n* (%) Hospital emergency for SUD	5 (2.2)	1 (0.7)	20 (9.2)	19.2	0.0001*
Withdrawal symptoms	14 (6.3)	6 (4.1)	53 (24.4)	46.0	0.0001*
Opiate consumption	2 (0.9)	4 (2.7)	28 (12.9)	32.5	0.0001*
Cocaine consumption	8 (3.6)	10 (6.8)	82 (37.8)	105.9	0.0001*
Alcohol consumption	28 (12.6)	23 (15.5)	125 (57.6)	125.9	0.0001*
Cannabis consumption	17 (7.6)	20 (13.5)	77 (35.5)	59.0	0.0001 *
Benzodiazepine consumption	8 (3.6)	8 (5.4)	47 (21.7)	43.4	0.0001 *
COVID-19-related variables, *n* (%)					
COVID-19 concern	132 (59.2)	87 (58.8)	123 (56.7)	0.3	0.854
COVID-19 symptoms	29 (13)	10 (6.8)	38 (17.5)	8.9	0.011
Positive for COVID-19	1 (0.4)	2 (1.4)	5 (2.3)	NA	NA
Broke the lockdown norm	54 (24.2)	44 (29.7)	108 (49.8)	34.0	0.0001 *
Working	38 (17)	29 (19.6)	33 (15.2)	1.2	0.549
Reduced income	56 (25.1%)	43 (29.1%)	71 (32.7%)	3.1	0.212
Change of address	21 (9.4)	8 (5.4)	20 (9.3)	2.2	0.326
Worsened family relationship	39 (17.5)	26 (17.6)	74 (34.3)	21.2	0.0001 *
Unresolved social matters	21 (9.5)	11 (7.5)	48 (22.4)	21.7	0.0001 *
Telematic treatment	92 (41.3)	78 (52.7)	67 (30.9)	17.6	0.0001 *
Self-reported impaired health	51 (22.9)	24 (16.2)	70 (32.3)	12.8	0.002
Positively valued treatment	176 (78.9)	113 (76.4)	137 (63.1)	15.2	0.0001 *
Psychiatric status, *n* (%)					
Insomnia symptoms	91 (40.8)	50 (33.8)	119 (54.8)	17.5	0.0001 *
Eating disorders symptoms	101 (45.5)	60 (40.5)	125 (57.6)	11.7	0.003
Alterations of sexual behaviour	29 (13)	19 (12.8)	37 (17.1)	1.9	0.392
Psychiatry emergencies	9 (4)	3 (2)	17 (7.8)	6.9	0.031
Anxiety symptoms (CAS)	105 (49.8)	65 (47.1)	163 (79.1)	50.2	0.0001 *
Panic attack	23 (10.3)	9 (6.1)	38 (17.5)	11.8	0.003
BPRS (above average 27)	75 (33.6)	49 (33.1)	130 (59.9)	39.2	0.0001 *
Depressive symptoms in BPRS	64 (28.8)	38 (25.7)	106 (48.8)	27.5	0.0001 *
CGI-S (grater severity, score 5–7)	16 (7.2)	8 (5.4)	45 (20.7)	27.1	0.0001 *
Worsening (global impression of change)	57 (25.7)	40 (27)	140 (64.5)	83.4	0.0001 *

BPRS, Brief Psychiatric Rating Scale; SUD, substance use disorder.

* Significant after Bonferroni correction

**Table 5:** Multivariate analysis: previous consumption pattern, COVID-19-related variables, SUD variables, and psychiatric status during lockdown

	Improved pattern versus worsened pattern				Maintained pattern versus worsened pattern				Improved pattern versus maintained pattern			
	Wald	sig.	OR	95% CI	Wald	sig.	OR	95% CI	Wald	sig.	OR	95% CI
**Model 1: prior to lockdow**n												
*Gender*												
Age (under/above average 47 years)	7.8	0.005	1.8	1.2–2.7								
Poly-SUDs	10.1	0.001	0.4	0.3–0.7					10.3	0.001	0.4	0.2–0.7
Injecting drug use												
Current CUD	10.6	0.001	0.5	0.3–0.7	15.4	<0.0001	2.7	1.6–4.4				

**During lockdown**												
**Model 2: COVID-19-related variables during lockdown**												
*Gender*												
Age (under/above average 47 years)	7.2	0.007	0.6	0.4–0.9								
Telematic treatment					7.3	0.007	0.5	0.3–0.8	5.1	0.024	0.6	0.4–0.9
Positively valued treatment	7.5	0.006	0.5	0.3–0.8								
Broke the lockdown norm	20.1	<0.001	2.7	1.7–4.1	9.4	0.002	2.1	1.3–3.3				
Worsened family relationship	4.6	0.032	1.7	1.0–2.7	4.5	0.035	1.8	1.0–3.1				
Unresolved social matters	7.7	0.005	2.3	1.3–4.2	7.6	0.006	2.7	1.3–5.6				

**Model 3: psychiatric status during lockdown**												
*Gender*												
Age (under/above average 47 years)	12.9	<0.001	2.1	1.4–3.2					4.1	0.042	1.6	1–2.4
Insomnia symptoms					4.1	0.04	1.6	1–2.6				
Anxiety symptoms (CAS)	14.2	<0.001	0.4	0.2–0.6	13.3	<0.0001	2.9	1.6–5				
BPRS (above average 27)	5.3	0.021	0.6	0.4–0.9								

## Appendix

Xarxa d'Atenció a les Drogodependències − Grup de Recerca sobre el COVID-19 (XAD-COVID-19-Group): Marta Perea Ortueta (Hospital Universitari Vall d'Hebron, Barcelona); Nieves Martínez-Luna (Hospital Universitari Vall d'Hebron, Barcelona); Francina Fonseca (Hospital del Mar, Barcelona); Susana Jornalé (CAS de la Mina, Barcelona); Xavier Samper (Hospital de Santa Maria, Lleida); Alicia Clariso (Hospital de Santa Maria, Lleida); Miriam Dueñas (CAS d'Horta-Guinardó, Barcelona); Armando Tirado (CAS d'Horta-Guinardó, Barcelona); Begoña Irala Indart (CAS Fontsanta, Cornellà de Llobregat); Rosa Pi i Marí (CAS Fontsanta, Cornellà de Llobregat); Víctor Martí Carrasco (CAS de Rubí, Rubí); Amalia Gual de Torrella (CAS de Rubí, Rubí); Francesco Panicali (CAS de Granollers, Granollers); Eulàlia Sabater Puig (Servei d'Addiccions i Salut Mental HUSJR, Reus); Tre Borràs i Cabacés (Servei d'Addiccions i Salut Mental HUSJR, Reus); Silvia Martínez (CAS del Prat, El Prat del Llobregat); Beth Pallejà i Martos (CAS del Prat, El Prat del Llobregat); Raúl Yuste (CAS del Baix Llobregat, Sant Feliu de LLobregat); Carolina Franco (CAS de Les Corts, Barcelona); Cinta Soler (CAS de Tortosa, Tortosa).
